# Foot tapping and unilateral vertical jump performance in athletes after knee surgery: an explorative cross-sectional study

**DOI:** 10.1186/s13102-022-00422-4

**Published:** 2022-03-03

**Authors:** Eduard Kurz, René Schwesig, Stefan Pröger, Karl-Stefan Delank, Thomas Bartels

**Affiliations:** 1 Department of Orthopedic and Trauma Surgery, Martin-Luther-University Halle-Wittenberg, Halle (Saale), Germany; 2 Joint Surgery Center, Sports Clinic Halle, Halle (Saale), Germany

**Keywords:** Knee joint, Anterior cruciate ligament, Rehabilitation, Return to sports

## Abstract

**Background:**

Guiding athletes through the rehabilitation process and judging the time at which return to sports can be enabled after anterior cruciate ligament (ACL) injuries are still challenging processes. The purpose of this explorative cross-sectional study was to retrospectively compare unilateral vertical jump as well as vertical foot tapping outcomes in athletes returned to sports after ACL reconstruction (ACLR) with uninjured athletes.

**Methods:**

Seven-teen ACLR athletes (male/female: 12/5) were examined 11 (6–23) months after their ACL injury and after return to sport clearance together with 67 uninjured athletes (male/female: 51/16). Seventeen age and stature matched controls were selected from the sample of uninjured athletes. Participants unilaterally performed acyclic (squat jump, SJ; drop jump, DJ) and cyclic (foot tapping, FT) tests. SJ peak power, DJ take-off efficiency (TOE) and FT coefficients (FTC) were compared between ACLR and matched as well as unmatched control groups. Limb symmetry index (LSI) as well as performance score were calculated.

**Results:**

Analyses of the SJ peak power revealed moderate effects of group (right: *P* < 0.09, η_p_^2^ = 0.06; left: *P* < 0.05, η_p_^2^ = 0.08). The TOE was largely affected by group (right: *P* < 0.01, η_p_^2^ = 0.12; left: *P* < 0.01, η_p_^2^ = 0.13). No effect of group was found on the FTC (*P* > 0.8, η_p_^2^ < 0.01). The SJ peak power LSI (*r* = 0.46, *P* < 0.07) and TOE LSI (*r* = 0.38, *P* = 0.13) were positively associated with the performance score of the ACLR group.

**Conclusion:**

Although already returned to sports, the ACLR group underperformed the matched and unmatched control groups significantly. Unilaterally performed vertical jumps may provide additional information on athletes’ rehabilitation progress and help to manage the rehabilitation process and decisions on potential readiness after ACLR. More attention should be paid to the direction of the LSI results.

## Background

Most athletes expect to return to their pre-injury sport without restrictions after a reconstruction of their torn anterior cruciate ligament (ACLR). Different indicators for a safe and successful return to sports have been identified [1]. However, guiding injured patients safely through the active rehabilitation process and releasing athletes as returned to sports are still challenging processes. Frequently, for different functional outcomes a certain degree of limb symmetry is recommended as a minimal prerequisite. Applying those criteria to a young and athletic population revealed only a low proportion (14%) of athletes who met all recommended thresholds [2] at the time of return to sports. Moreover, recent results revealed that indicators of absolute performance were superior to limb symmetry when judging athlete’s readiness after ACLR [3, 4]. This encourages using both absolute performance and limb symmetry measures to support the decisions, especially when pre-injury results are not available.

To characterize lower extremity performance, different vertical or horizontal jump or hop tests are routinely used and accepted as a cost-efficient and practicable option to evaluate athletes’ leg power capacity [5]. Of those, the squat jump (SJ) and the drop jump (DJ) are considered to provide evidence on athletes’ primarily concentric or eccentric dynamic vertical performance ability. The bilateral execution of the SJ and DJ tests are more common, which in turn hampers judgement of unilateral performance. The unilateral alternative can be useful for comparative purposes to judge readiness after a sustained injury. Unilateral SJ was already used to determine functional asymmetries in ACLR participants [6–9].

To address the efficiency of the fast stretch–shortening cycle, various forms of the DJ are frequently applied within strength and conditioning programs. Three different execution strategies are reported in the literature: minimize ground contact time (bounce DJ), maximize jumping height (countermovement DJ), and a mixed strategy in which both demands are considered [10]. From the perspective of talent identification as well as fast stretch–shortening cycle conditioning, the first strategy would probably be the best choice [11]. However, for general conditioning purposes with multiple DJs the second strategy is primarily implemented. The mixed strategy is most commonly applied for performance analyses.

Most recommendations to judge rehabilitation progress or release athletes for a return to sports combine different tests which are acyclic in nature. This aspect was criticized by Maulder and Cronin [12], who encouraged clinicians to use tests examining different qualities of performance. Especially when protocols are applied independently of type of sports or disciplines, a broader range of fundamental performance requirements could add beneficial information. One established cyclic neuromuscular performance test is the vertical foot tapping test [13]. The vertical foot tapping test examines the cyclic speed performance under loaded condition of the lower extremities. Foot tapping tests are considered to be independent of strength qualities and therefore a valuable supplement to strength-related performance tests.

Therefore, the primary purpose of this investigation was to compare performance outcomes of athletes returned to sports after ACLR with uninjured athletes utilizing unilateral vertical jump as well as vertical foot tapping tests. We hypothesized that the ACLR athletes would show performance deficits in the acyclic as well as the cyclic performance tests. The secondary aim was to relate overall performance to symmetry in both groups. We expected a positive association between overall performance and limb symmetry.

## Methods

### Study design and participants

Eighty-four athletes (handball: 33, soccer: 25, volleyball: 15, ice hockey: 5, track and field: 3, judo: 2, swimming: 1) were included in this cross-sectional study. To be eligible, the following criteria had to be met: no musculoskeletal injury or surgery at the lower extremities in the past 6 months, no vestibular, visual, or hearing impairments. Participants were excluded from the analyzes if they failed to successfully complete any of the required cyclic or acyclic performance tests. The participant characteristics are detailed in Table 1. Athletes’ activity level before injury was assessed using the 11-level Tegner scale [14]. The study population comprised 67 uninjured athletes and 17 athletes after completing their rehabilitation phase following their ACLR surgery performed by the same experienced surgeon (TB, hamstring graft) and return to sports clearance 11 (6–23) months after their ACL injury. Eleven (65%) ACLR participants had dominant (limb used to kick a ball) side injuries.

**Table 1:** Demographic characteristics of the participants presented as means with standard deviations and minimum and maximum

	ACLR athletes (n = 17)	Uninjured athletes (n = 67)
Male/female	12/5	51/16
Age (years)	25 (7, 15–39)	23 (4, 15–35)
Height (m)	1.80 (0.09, 1.65–1.98)	1.84 (0.10, 1.63–2.06)
Mass (kg)	78 (15, 50–100)	81 (12, 61–108)
BMI (kg/m^2^)	24.1 (3.5, 17.4–31.4)	23.8 (2.1, 19.7–29.0)
Breakdown of number of athletes from each sport, n (%)		
Handball	5 (29)	28 (42)
Soccer	8 (47)	17 (25)
Volleyball	1 (6)	14 (21)
Ice hockey		5 (7)
Track and field	1 (6)	2 (3)
Judo	2 (12)	
Swimming		1 (1.5)

*ACLR* anterior cruciate ligament reconstruction, *BMI* body mass index

Seventeen age and stature matched controls (mCON) were selected from the larger sample of uninjured athletes. Their sides were assigned according to the injured side of their matched peers. The subgroup of mCON (n = 17) was on average 24 (SD 4, 18–31) years old (height: 1.83 (SD 0.11, 1.65–1.97) m, mass: 82 (SD 15, 61–102) kg, BMI: 24.5 (SD 2.5, 19.7–29.0) kg/m^2^). This investigation was approved by the local ethics committee (approval number: 2016-144) and complied with the Declaration of Helsinki.

### Performance tests

Participants performed acyclic (unilateral vertical jump tests) as well as cyclic (15 s vertical alternating foot tapping (FT) test [15]) tests after a standardized warm-up of 15-min duration (running on a treadmill, a series of bilateral and unilateral jumping drills). Two different types of vertical jumps were performed unilaterally. For the squat jump (SJ), the participant started in a static, semi-squatting position and no preparatory countermovement was allowed. Thus, during SJ a concentric, shortening contraction type (explosive strength) was executed. For the drop jump (DJ), in contrast, the participant had to utilize the fast stretch–shortening cycle (reactive strength). After stepping off a 30 cm box, the athlete dropped down with the instructions to keep the ground contact time as short as possible and to reach maximum possible jumping height (mixed strategy [10]) and without the heel touching the ground. Especially for the bilaterally executed DJ, the participants are instructed to jump down to a distance of half the individual’s body height [16]. Here, the box was located approximately 30 cm away from the target on the floor to ensure a safe single-leg DJ execution. For each jump test, participants performed 1 familiarization trial followed by 2 attempts used for further evaluation. Each repetition was performed with at least 10 s rest. The FT test was performed once. To ensure comparability, participants were asked to keep their hands akimbo throughout the entire tests.

### Measurement device

For each test performed, the ground contact times were measured by the contact plates at the center of a SpeedCourt device (GlobalSpeed GmbH, Hemsbach, Germany). This device was implemented for conditioning purposes in healthy and athletic populations [17] as well as in injured athletes aiming to return to pre-injury activities [18]. Flight times (from take-off to landing) obtained by the device were used to calculate jumping heights according to the formulas outlined in Komi and Bosco [19].

### Performance outcomes

Best SJ height was used for further analyses. For the SJ, peak power was additionally estimated from jumping height and body mass using the equation developed and validated by Sayers et al. [20].

To determine best DJ performance, the 2 outcomes ‘ground contact time’ and ‘jumping height’ need to be taken into account. Therefore, take-off efficiency (TOE, Eq. 1) was calculated according to Ambarov et al. [21]. This unitless quantity quantifies the speed strength performance, specifically the reactive strength capacity. The DJ attempt with the best TOE was used for further analyses.

1 $$TOE=\frac{{flight\,time\,(s)}^{2}}{ground\, contact\, time \,(s)}$$

For the FT test, the ground contact times obtained were averaged separately for each side. The sum of left and right sided contacts were used to determine the FT frequency. Participants’ foot tapping performance was quantified as an FT coefficient (FTC, Eq. 2) as introduced by Voss and colleagues [11]. A higher FTC corresponds to a higher cyclic performance capacity.

2 $$FTC=\frac{foot\, tapping\, frequency\, (Hz)}{ground \,contact \,time \,(ms)}\times 100$$

Limb symmetry indices (LSI, Barber et al. [22]) were calculated for SJ height, SJ peak power, DJ height, DJ TOE as well as the averaged FT ground contact times. Some athletes’ injured sides revealed a better performance, resulting in LSI values greater than 100 percent (cf. [18]). Thus, those results were direction corrected (the smaller value was divided by the larger value [23]) before comparing LSI values statistically.

### Overall performance

The results of the five tests (right and left sided SJ peak power and DJ TOE as well as FTC) were converted into z-scores based on the whole sample of 84 athletes. Z-scores indicate how many standard deviations an individual’s score is away from the mean. Accordingly, a z-score of zero equals the sample’s mean. Athlete’s z-scores were summed-up resulting in the performance score (PS), which indicates overall performance.

### Statistical analysis

Statistical analyses were carried out with SPSS Statistics 28 (IBM, Armonk, NY, USA) software for Windows. Comparisons of demographic characteristics and symmetry (LSI) between groups were conducted using either independent Student’s t tests or Mann–Whitney U tests. To assess subgroup effects on performance outcomes (SJ jumping height, SJ peak power, TOE, FTC) separate univariate analyses of variance were computed. Post-hoc tests on subgroup were performed utilizing the least significant differences test. Statistical significance was set at *P* < 0.05. Practical relevance was estimated calculating partial eta squared (η_p_^2^) with values ≥ 0.01, ≥ 0.06, ≥ 0.14 indicating small, moderate, or large effects, respectively. Further, linear associations between time since ACL injury and different performance outcomes as well as the overall performance (summed-up z-scores) and LSI values were examined using Pearson’s product moment correlation. To unravel possible relationships of the performance outcomes with the level of activity (Tegner scores), the Spearman’s rank correlation coefficient was used.

## Results

### Participant characteristics

All participants examined had a median Tegner activity level of seven, which corresponds to a high and competitive activity level. The ACLR athletes did not differ from the selected matched control subgroup concerning their age and their anthropometric characteristics (*P* > 0.4) as well as their Tegner activity level (*U* = 137.0, *z* =  − 0.32, *P* > 0.7, effect size =  − 0.06). Two participants (male/female: 1/1) sustained a contact injury, whereas 15 (88%) participants had a non-contact ACL injury. Fourteen (82%) participants performed team sports (soccer: 8, handball: 5, volleyball: 1). Two (12%) participants have practiced judo and one (6%) female participant was a javelin thrower.

### Unilateral vertical jump performance

The ACLR limb achieved lower jumping heights compared with their contralateral side as well as both uninjured subgroups. Detailed results on SJ or DJ jumping heights, SJ peak power or DJ take-off efficiency are given in Table 2. The SJ peak power estimations revealed moderate effects of group (injured, matched or right sides: *F* (2, 81) = 4.4, *P* < 0.02, η_p_^2^ = 0.098). Subsequent post-hoc comparisons indicated differences between the ACLR group and both the matched as well as unmatched control groups (*P* < 0.02) but not between the control subgroups (*P* > 0.8). The take-off efficiency of the DJ was largely affected by group (injured, matched or right sides: *F* (2, 81) = 11.9, *P* < 0.001, η_p_^2^ = 0.227). Post-hoc comparisons indicated differences between ACLR and matched as well as unmatched controls (*P* < 0.001), respectively.

**Table 2:** Participants’ jumping heights (cm), squat jump peak power (W) and drop jump take-off efficiency (unitless) presented as means with 95% confidence intervals

	ACLR athletes (n = 17)	Uninjured athletes (n = 67)		*P* value^a^
		mCON (n = 17)	uCON (n = 50)	
SJ height				
Injured	13.7 (11.1–16.3)			< 0.001
Matched		20.0 (18.1–22.0)		
Right			20.4 (19.2–21.6)	
SJ height				
Uninjured	17.3 (14.9–19.8)			0.03
Matched		20.6 (18.4–22.8)		
Left			21.0 (19.6–22.4)	
DJ height				
Injured	13.1 (10.7–15.4)			< 0.001
Matched		17.5 (15.9–19.2)		
Right			17.9 (16.6–19.2)	
DJ height				
Uninjured	17.3 (15.8–18.8)			0.48
Matched		17.6 (15.7–19.4)		
Left			18.4 (17.3–19.6)	
SJ peak power				
Injured	2320 (1906–2735)			< 0.02
Matched		2882 (2499–3264)		
Right			2842 (2679–3005)	
SJ peak power				
Uninjured	2545 (2146–2944)			0.18
Matched		2914 (2515–3313)		
Left			2875 (2708–3042)	
DJ take-off efficiency				
Injured	0.35 (0.28–0.43)			< 0.001
Matched		0.59 (0.52–0.66)		
Right			0.59 (0.53–0.64)	
DJ take-off efficiency				
Uninjured	0.50 (0.45–0.55)			0.16
Matched		0.57 (0.50–0.64)		
Left			0.59 (0.54–0.64)	

*ACLR* anterior cruciate ligament reconstruction, *DJ* drop jump, *mCON* matched controls, *uCON* unmatched controls, *SJ* squat jump

^a^*P* value of univariate analysis of variance

### Vertical foot tapping performance

**Fig. 1:**
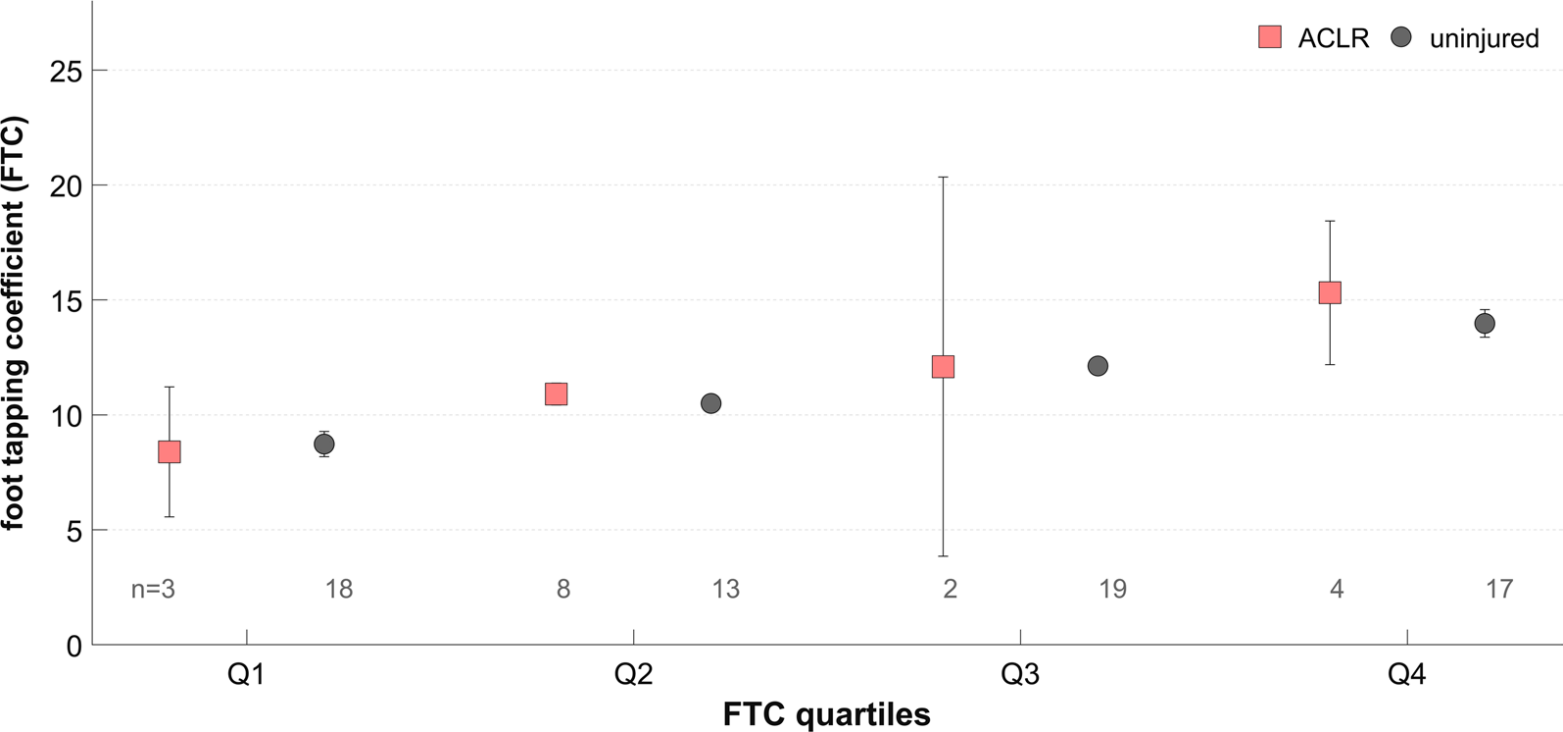
Results of foot tapping coefficients (FTC) of ACLR (n = 17) and uninjured (n = 67) athletes separated into quartiles. Values presented as mean with 95% confidence intervals

There was no effect of group on the FTC (*F* (2, 81) = 0.1, *P* > 0.8, η_p_^2^ < 0.01). On average, the ACLR athletes (11.6, SD 2.6), the matched (11.4, SD 2.3) and unmatched (11.3, SD 2.2) controls performed equally on the cyclic neuromuscular test. Thus, the FTC results of all participants examined were classified into quartiles (Fig. 1). The results showed that ACLR athletes can be found within each quartile. Furthermore, for ACLR athletes the FTC was positively related to the Tegner activity level (ρ = 0.54, *P* < 0.03). Matched controls’ Tegner activity level, in contrast, was not associated with the FTC results (ρ = 0.21, *P* > 0.4).

### Limb symmetry

**Fig. 2:**
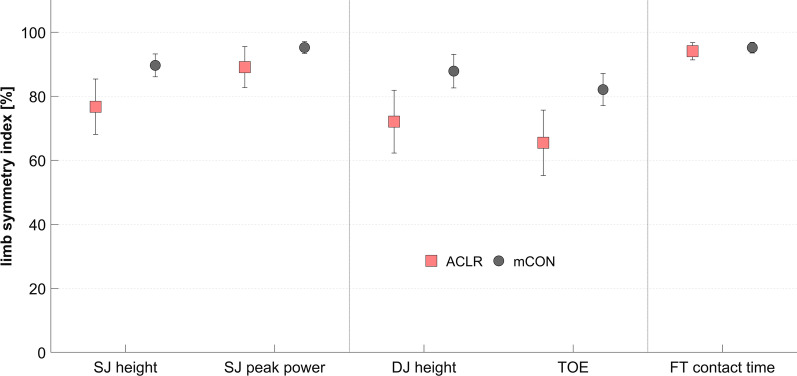
Direction-corrected limb symmetry indices of ACLR athletes (n = 17) and the matched controls (mCON, n = 17) of squat jump (SJ, jumping height and peak power), drop jump (DJ, jumping height and take-off efficiency, TOE) and foot tapping (FT) contact time outcomes. Values presented as mean with 95% confidence intervals

For the SJ height, SJ peak power and TOE, 1 ACLR athlete and 9 matched controls showed values greater than 100. Three ACLR athletes and 10 matched controls revealed values greater than 100 for the DJ height. For the FT contact times, 5 ACLR athletes and 5 matched controls showed values above 100. The results of the corrected LSI values are displayed in Fig. 2. The direction-corrected LSI values of SJ performance revealed a higher asymmetry in ACLR athletes as compared with the matched controls (*P* < 0.02, Mann–Whitney U test). A higher asymmetry between sides in the ACLR group was also found for the DJ outcomes (*P* < 0.01, independent Student’s t test). No differences could be verified for the direction-corrected LSI values of FT contact times (*P* > 0.7, Mann–Whitney U test).

**Fig. 3:**
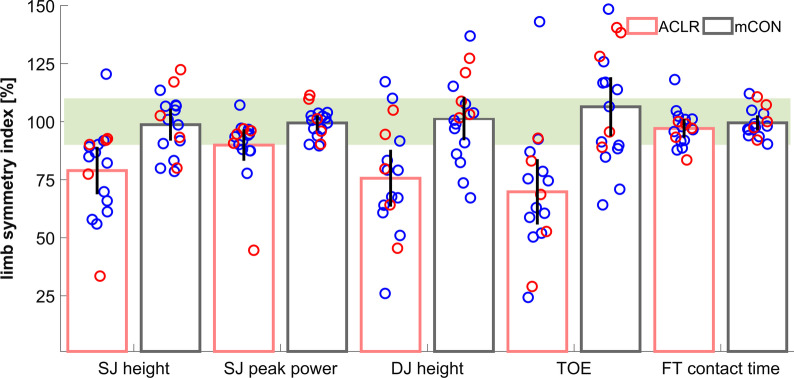
Individual uncorrected LSI values of the ACLR athletes (n = 17) and the matched controls (mCON, n = 17) with mean and 95% confidence intervals of squat jump (SJ, jumping height and peak jumping power), drop jump (DJ, jumping height and take-off efficiency, TOE) and foot tapping (FT) contact time outcomes. The green area represents the cutoff region for LSI values

Sixteen (94%) ACLR athletes revealed at least one LSI value below the 90% or above the 110% thresholds. The uncorrected LSI values within the 5 unilaterally examined outcomes are displayed in Fig. 3.

### Overall performance

Overall performance score (PS) was largely affected by group (*F* (2, 81) = 5.5, *P* < 0.01, η_p_^2^ = 0.12). Post-hoc comparisons indicated differences between the ACLR group and the matched (*P* = 0.009) as well as unmatched control group (*P* = 0.002). The direction-corrected SJ peak power LSI (*r* = 0.46, *P* < 0.07) and DJ TOE LSI (*r* = 0.38, *P* = 0.13) was positively associated with the performance score of the ACLR group but not with the performance score of the matched control group (*P* > 0.4).

### Inter-relationships

**Fig. 4:**
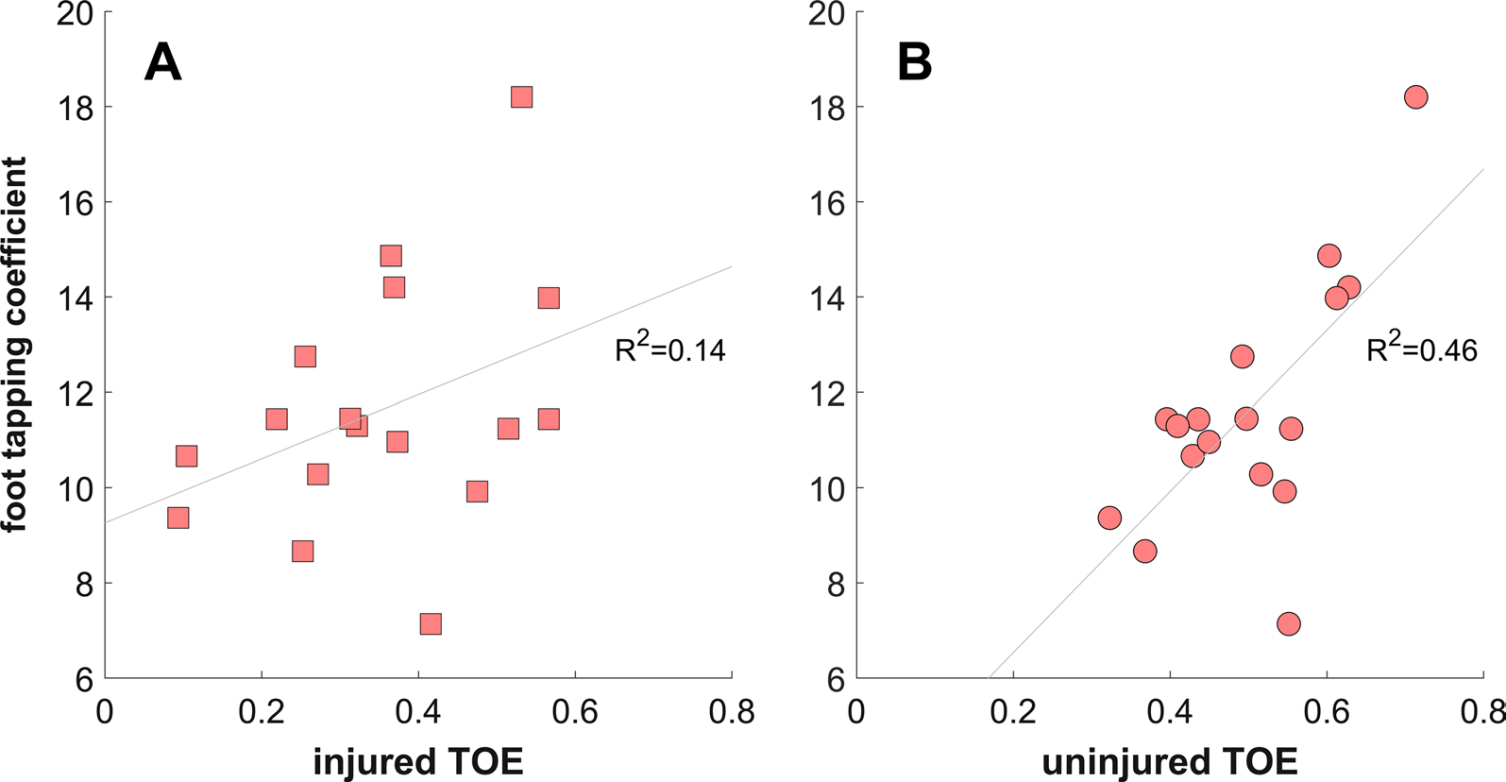
Association between the drop jump take-off efficiency (TOE) and the foot tapping coefficients at the injured (**A**) and uninjured (**B**) sides of the ACLR athletes (n = 17)

Time since injury was not associated with either performance outcome (*P* > 0.1, − 0.4 < r < 0.4). For the ACLR group, the FTC was positively associated with the SJ height (*r* = 0.50, *P* < 0.05), SJ peak power (*r* = 0.50, *P* < 0.04), DJ height (*r* = 0.65, *P* < 0.01) and TOE (*r* = 0.68, *P* < 0.01, Fig. 4) at the uninjured side but not with the results at the injured side (*P* > 0.06). Interestingly, no such relationships could be verified for the matched control group at either side.

## Discussion

This study investigated lower extremity functional performance of acyclic as well as cyclic demands in ACLR athletes who were already released for return to sports. Overall performance as well as limb symmetry was lower in ACLR athletes. Cyclic performance as depicted by the 15 s vertical foot tapping test was comparable between groups. Unilateral vertical jump performances were markedly lower in the ACLR group. Symmetry of the unilateral vertical jumps correlated at least weakly with overall performance in ACLR athletes only.

### Unilateral vertical jump performance

To quantify deficits of specific strength qualities, squat, countermovement and drop jumps are most commonly applied in a bilateral fashion. In addition to generally used horizontal hop tests in ACLR subjects, Hopper and colleagues [6] employed a vertical jump unilaterally to enable comparisons between sides. Their flight times recorded on a force plate from reconstructed (0.21–0.44 s) and uninjured (0.31–0.47 s) legs are comparable to those recorded with our device (injured: 0.20–0.43 s, uninjured: 0.23–0.45 s). Pairot de Fontenay et al. [8] examined a comparable sample of ACLR athletes (11 males) cleared for return to sports. They found a 24% lower jumping height at the injured side. Our male athletes revealed a 20% lower jumping height on average at the injured side. However, as can be seen in Fig. 3, one athlete reached a 21% higher jumping height on his injured side. This finding underpins the direction specificity of the LSI values, which the clinician must keep in mind. The ‘direction of asymmetry’ was emphasized by Impellizzeri et al. [24] and Maloney [25]. From a practical perspective it may not be irrelevant which limb performed better or worse.

Since reactive strength qualities are basic requirements for jumping, hopping, or running activities, injured athletes need to address the reactive capacity throughout the rehabilitation process. The DJ outcomes revealed the largest differences between subgroups. Single-leg reactive neuromuscular performance was affected most by the ACL injury. After nine months post surgery, King et al. [26] found smaller side effects in front hop performance as compared with unilateral DJ. They concluded that at that rehabilitation stage the horizontal-type hop test may overestimate the actual functional status. In contrast to the front hop, the DJ executed with an immediate vertical take-off utilizes the fast stretch–shortening cycle. The jumping strategy used to absorb the impact forces during DJ execution results in shorter or longer ground contact times. With shorter ground contact times, the leg and ankle stiffness will increase [27] and therewith the TOE. Despite the fact that lower extremity stiffness is associated with athletic performance, higher peak forces bear risks for cartilage overload or bony injuries [28]. Investigating DJ kinematics, uninjured female athletes showed different landing patterns [29]. Stiff, knee or hip dominant landing strategies were distinguished according to degrees of joint flexion demonstrated. Lower hip and knee joint flexion landing angles (greater dominance) were found for bilateral versus unilateral DJ executions. This may indicate more effective force absorption in double-legged DJ. Consequently, after a sustained ACL injury, one useful strategy might be to realize larger joint moments [30] to compensate for vertical impacts at the expense of stretch–shortening cycle efficiency. This leads to longer ground contact times and limited TOE. Therefore, TOE can be interpreted as a surrogate for lower extremity stiffness.

### Vertical foot tapping performance

Tapping tests are routinely applied in clinical environments to assess neurological dysfunction in upper and lower extremities [31]. In professional sports, tapping tests examine cyclic neuromuscular performance and are used for talent identification [11]. Although lower extremities’ cyclic performance was on average similar between injured and uninjured athletes, the results of the ACLR participants corresponded to their Tegner activity level. However, due to the relatively long duration of the FT test (15 s), the individual’s anaerobic capacity very likely influenced the results [15]. In ACLR athletes, each of the unilateral vertical jump performance outcomes was moderately correlated with the FTC, but only at the uninjured side. This suggests that the ACL injury affects the person as a whole, and different qualities of performance are less independent from each other in injured athletes.

### Limb symmetry and overall performance

Only the acyclic performance outcomes revealed substantial side-to-side differences, which were larger for the ACLR athletes. Interestingly, for TOE only 1/17 ACLR athletes and 4/17 matched controls reached the threshold value of 90%. Some concerns exist surrounding the utilization of LSI values as the only discharge criteria. Wellsandt et al. [3] followed 70 ACLR patients, eleven of whom sustained a second injury, and found that the estimated pre-injury capacity was more sensitive in predicting a second injury than the LSI values. In another study, 64 ACLR patients were followed up to 2 years after completing the rehabilitation program [4]. Although all patients finished the program with LSI values > 90%, at the end of rehabilitation only 46 successfully returned to their pre-injury level. Compared to those patients who failed to return, they showed higher absolute scores on all hop tests performed.

### Limitations and future directions

The participants in this study were recruited from a rehabilitation center and completed routine performance measures, best representing a real-life cohort. Consequently, this study is limited to a relatively small and heterogeneous sample. Since acute fatigue was shown to alter intrinsic risk factors in non-contact ACL injured participants [32], future studies should elaborate effects of different protocols as well as task specificity. In this context, in particular the execution strategies of the drop jumps should be considered more consistently in assessments of athletes’ readiness. Besides quantitative evaluations, movement quality ratings (e.g., drop jump landing) would complement the competency profile.

## Conclusion

Based on the tests utilized in this study, the ACLR group underperformed the matched and unmatched control groups significantly, although already returned to sports. Unilaterally performed vertical jumps may provide additional information on athletes’ rehabilitation progress and therefore help to manage the rehabilitation process and decisions on potential readiness after ACLR. More attention should be paid to the direction of the LSI results.

## Data Availability

The datasets used and analyzed during the current study are available from the corresponding author on reasonable request.

## Abbreviations

ACL: Anterior cruciate ligament
ACLR: Anterior cruciate ligament reconstruction
DJ: Drop jump
η_p_^2^: Partial eta squared
FT: Foot tapping
FTC: Foot tapping coefficient
LSI: Limb symmetry index
mCON: Matched controls
PS: Performance score
SJ: Squat jump
TOE: Take-off efficiency
uCON: Unmatched controls
